# A Novel Calibration Step in Gene Co-Expression Network Construction

**DOI:** 10.3389/fbinf.2021.704817

**Published:** 2021-11-23

**Authors:** Niloofar Aghaieabiane, Ioannis Koutis

**Affiliations:** Department of Computer Science, New Jersey Institute of Technology, Newark, NJ, United States

**Keywords:** Gene co-expression networks, Similarity function, Clustering, Gene Ontology, Topological Overlap Measure

## Abstract

High-throughput technologies such as DNA microarrays and RNA-sequencing are used to measure the expression levels of large numbers of genes simultaneously. To support the extraction of biological knowledge, individual gene expression levels are transformed to Gene Co-expression Networks (GCNs). In a GCN, nodes correspond to genes, and the weight of the connection between two nodes is a measure of similarity in the expression behavior of the two genes. In general, GCN construction and analysis includes three steps; 1) calculating a similarity value for each pair of genes 2) using these similarity values to construct a fully connected weighted network 3) finding clusters of genes in the network, commonly called modules. The specific implementation of these three steps can significantly impact the final output and the downstream biological analysis. GCN construction is a well-studied topic. Existing algorithms rely on relatively simple statistical and mathematical tools to implement these steps. Currently, software package WGCNA appears to be the most widely accepted standard. We hypothesize that the raw features provided by sequencing data can be leveraged to extract modules of higher quality. A novel preprocessing step of the gene expression data set is introduced that in effect calibrates the expression levels of individual genes, before computing pairwise similarities. Further, the similarity is computed as an inner-product of positive vectors. In experiments, this provides a significant improvement over WGCNA, as measured by aggregate *p*-values of the gene ontology term enrichment of the computed modules.

## 1 Introduction

The availability of high-throughput technologies like DNA microarrays (Reshef et al., 2011) or RNA-sequencing (Hrdlickova et al., 2017) (RNA-seq) has motivated several approaches for developing a computational understanding of genes and their functionalities. A prominent example are gene co-expression networks (GCNs) that are used to perform tasks such as functional annotations (Serin et al., 2016; Ma et al., 2018), biological process (Emamjomeh et al., 2017), pathway analysis (Ma et al., 2018; van der Wijst et al., 2018), and disease mechanism understanding (Parsana et al., 2019). In a GCN, nodes correspond to genes, and the weight of the connection between two nodes is a measure of similarity in the expression behavior of the two genes (Tieri et al., 2019).

In general, given a gene expression data set (provided by DNA microarray or RNA-seq) a GCN pipeline includes the following steps; *1-Similarity*: Calculation of a similarity value for each pair of genes, *2-Adjacency*: Further processing of these similarity values to construct a network encoded by its adjacency matrix, *3-Clustering*: Computation of clusters of genes in the network, commonly called *modules* (Schaefer et al., 2017; van Dam et al., 2017), and *4-Evaluation*: Evaluation of the modules based on measuring their *enrichment* with Gene Ontology (GO) terms (Khatri and Drǎghici, 2005). Modules can later divulge significant biological intuition.

The specific implementation of these steps can significantly impact the final output and the downstream biological analysis. In particular, the similarity and adjacency steps can be implemented in various ways. For example, framework Petal (Petereit et al., 2016) instantiates them as follows: 1) *Similarity*: Computation of the Spearman correlation, 2) *Adjacency*: Construction of an initial network using the signum function and further modification so that it follows certain scale-free and small-world criteria (Barabási and Albert, 1999). On the other hand, WeiGhted Correlation Network Analysis (WGCNA) which is the most widely acceptable framework for GCN construction takes the following steps: 1) *Similarity*: Computation of the Pearson correlation, 2) *Adjacency*: Conversion of the negative correlation values into positive, further powering the coefficients so that the resulting network follows the scale-free criteria and adding information about second-order neighborhoods of the network, in the form of what is called the Topological Overlap Measure (TOM) of the network (Zhang and Horvath, 2005; Langfelder and Horvath, 2008).

GCN construction and analysis is well studied, for over a decade. But given its widespread use and applicability, the possibility of improving existing frameworks is tantalizing and motivates further research. We hypothesize that the raw features provided by sequencing data can be leveraged to extract modules of higher quality. To this end, we introduce a novel step that precedes the steps of the standard pipeline and is performed directly on the gene expression data set. This is a further processing of the level of the expression provided by the DNA microarrays: this in effect calibrates the expression levels of individual genes, before computing pairwise similarities. Further, we deviate from standard frameworks that use statistical measures for the similarity computation (Liu, 2017), and instead use a geometric measure, cosine similarity. Specifically we compute similarity as a simple inner-product of vectors of positive numbers. This is appropriate for our context, since expression levels are positive numbers, and avoids complications related to the interpretation of negative coefficients that are artificially inserted in the analysis via correlation measures. While simple, these steps have not been considered in earlier literature, to the best of our knowledge. As WGCNA appears to be the most widely accepted standard, we implement the proposed steps as modifications to the WGCNA framework, so that they can be easily incorporated into the current GCN construction and analysis workflow. The rest of the process for network construction is the same with WGCNA, to make things comparable. In multiple experiments, our modifications seem to provide an overall significant improvement over WGCNA on real data, as measured by aggregate *p*-values of the gene ontology (GO) term enrichment of the computed modules. Specifically, we run a set of experiments on six different data sets with sample sizes between 44 up to 438 and we found that in all but one cases, calibration combined with geometric similarity results in more enriched modules.

## 2 Methods

### 2.1 Proposed Steps

We describe the two novel steps that constitute our proposed modification to the standard pipeline.

#### 2.1.1 Calibration Step

Let *G* be an *m* × *n* gene expression matrix where *m* and *n* are the number of samples and genes respectively, and the entry *g*_*i*,*j*_ is the value of the expression gene *j* in sample *i*, as shown in Eq. 1.

$$G=\left(\begin{array}{cccc} g_{1,1} & g_{1,2} & \ldots & g_{1,n}\\ g_{2,1}, & g_{2,2} & \ldots & g_{2,n}\\ \vdots & \vdots & \ddots & \vdots \\ g_{m,1} & g_{m,2} & \ldots & g_{m,n} \end{array}\right)$$ (1)

In the calibration step, we filter the raw level of the expressions provided in *G*. Concretely, let *G*_*j*_ denote the *j*th column of *G* that contains the expression of gene *j*. Also define *μ*_*j*_ and $\sigma_{j}^{2}$ as the mean and variance of gene vector *G*_*j*_. Then for every gene *j* and sample *i* we calculate a calibrated expression *s*_*i*,*j*_ as follows:

$$s_{i,j}=\frac{1}{1+\exp\left(-\frac{1}{\sigma_{j}^{2}}\left(g_{i,j}-\mu_{j}\right)\right)}$$ (2)

It should be noted that *s*_*i*,*j*_ > 0. In the sequel, we denote by *S* = [*s*_*i*,*j*_] the gene expression matrix after the calibration step, and *S*_*j*_ the *j*th column of *S*.

#### 2.1.2 Similarity

We consider two variants of a similarity measure based on computing simple inner products between positive vectors.

In the first variant, we initially set *S*′ = *S* ^*T*^ *S*. Note that $s_{i,j}^{\prime }$ is the inner product between the calibrated expression levels of genes *i* and *j*. These similarity values $s_{i,j}^{\prime }$ may not be in the interval (0, 1). Therefore, in order to compute similarity values in the range (0, 1) we compute the final similarities *m*_*i*,*j*_ via the following normalization:

$$m_{i,j}=\frac{s_{i,j}^{\prime }-\underset{i,j}{\min}}{\underset{i,j}{\max}-\underset{i,j}{\min}}$$ (3)

where $\underset{i,j}{\min}$ and $\underset{i,j}{\max}$ denote the minimum and maximum entry over row *i* and column *j* of *S*′.

In the second variant, we let

$$m_{i,j}=\frac{S_{i}^{T}S_{j}}{\|S_{i}{\|}_{2}\|S_{j}{\|}_{2}}$$ (4)

where *S*_*i*_ denotes the *i*th column of *S*, and ‖ ⋅‖_2_ denotes the Euclidean norm of a vector. This is precisely the cosine similarity between the two vectors *S*_*i*_ and *S*_*j*_.

In both variants we have *m*_*i*,*j*_ = *m*_*j*,*i*_ and 0 < *m*_*i*,*j*_ < 1.

### 2.2 Adjacency

As we discussed earlier, the main goal of this study is to compare the effectiveness of the proposed steps with WGCNA. Let us summarize the WGCNA pipeline:

#### 2.2.1 WGCNA

1) Calculate the Pearson correlation on gene expression.
2) Convert the negative values to positive using Eq. 3.
3) Power the similarity matrix (element-wise) so that the network becomes scale-free.
4) Add topological information (TOM) to the network using Eq. 5.

Two remarks are due here.

1) A network is scale-free if the degree of its nodes follow a power law *p*(*k*) ∼ *k* ^−Γ^ where *k* is a non-negative real number. The scale-freeness criteria of a network can be measured using the *R* ^2^ fitting index of the linear model of   log (*p*(*k*)) that regresses on log(*k*). If *R* ^2^ approaches 1, then the scale-freeness criteria holds for the network.
2) T1he topological overlap measure (TOM) calculates the weight *ω*_*i*,*j*_ between genes *i* and *j* in the adjacency matrix by including second-order neighborhood information in gene interactions. For instance, if for two genes *i* and *j* there are multiple genes *k* showing a strong interaction with both *i* and *j*, then that adds extra strength in the weight *ω*_*i*,*j*_. More formally the weight is given in Eq. 5 (Zhang and Horvath, 2005).

$$\omega_{i,j}=\frac{l_{i,j}+a_{i,j}}{\min\left(k_{i},k_{j}\right)+1-a_{i,j}}$$ (5)

where *l*_*i*,*j*_ = *∑*_*u*_ *a*_*i*,*u*_ *a*_*u*,*j*_, and *a*_*i*,*j*_ is the similarity value between gene *i* and *j* from previous step, and *k*_*i*_ = *∑*_*u*_ *a*_*iu*_ is the degree of node *i*.

### 2.3 Calibration-Based Pipeline Variants

We now describe three pipelines for constructing a network from the raw expression data. They all use steps described in Sections 2.1, 2.2. We name the variants and specify them as follows:

#### 2.3.1 Alpha

1) Apply the calibration step and calculate matrix *S* according to Eq. 2.
2) Compute similarities according to Eq. 3.
3) Power the similarity matrix so that the network becomes scale-free.

#### 2.3.2 Beta

1) Apply the calibration step and calculate matrix *S* according to Eq. 2.
2) Compute similarities according to Eq. 4.
3) Power the similarity matrix so that the network becomes scale-free.

#### 2.3.3 Gamma

1) Follow steps 1-3 of Beta.
2) Add TOM to the network, according to Eq. 5.

All three variants include the calibration step and will be compared against the standard pipeline of WGCNA. We include Alpha to contrast it with the pure cosine similarity measure used in Beta and Gamma. Gamma includes TOM and its comparison with Beta shows that including second-order neighborhood information remains an effective tool in synergy with our proposed steps.

### 2.4 Clustering

Several algorithms for detecting modules in the network have been proposed; among them hierarchical clustering, partitioning, and neural networks have received the most attention (van Dam et al., 2017). In this study we used the “Dynamic Tree Cut” (Langfelder et al., 2007) package in R (R Core Team, 2013), which is the *de facto* standard and used with WGCNA. Dynamic Tree Cut is a version of hierarchical clustering that dynamically cuts the dendrogram depending on its shape which results in more flexibility in cluster identification. The authors have suggested that their method is capable of identifying nested clusters, and the resulting modules are more enriched with known GO (Langfelder et al., 2007).

## 3 Data, Evaluation and Results

In this section we discuss the evaluation of our three calibration-based pipelines and their comparison against WGCNA. We use six real datasets. For each dataset, we compute modules with the four different pipelines and then compare their quality. The only differentiation in these four different computations is in the construction of the network, as described in the previous section, and all other steps remain the same as in WGCNA.

### 3.1 Data Sets

The gene expression data sets have been downloaded from NCBI Gene Expression Omnibus GEO (Barrett et al., 2012). They are distinguished by their unique GEO Series (GSE) number. The first data set is the gene expression data of *Drosophila melanogaster* GSE34400 (Lundberg et al., 2012), and it contains 44 samples. The second data set is the gene expression data of kidney transplantation in human being patients GSE1291666 (Van Loon et al., 2019), and it contains 212 samples. The third data set is the gene expression data of transcriptional consequences of pharmacologic PPAR a, d, and g agonist administration in murine liver, heart, kidney, and skeletal muscle in *Mus musculus* organism GSE27948^1^, and it contains 300 samples. The fourth data set is the gene expression data of PAXgene allergic asthma patients at baseline GSE13739 (Choy et al., 2016) and it contains 309 samples. The fifth data set is the gene expression data of livers of F2 mice (C57BL/6 X DBA/2) deficient in leptin receptor (db/db) of *Mus musculus* GSE30140 (Davis et al., 2012) and it contains 435 samples. The last (sixth) data set is the gene expression data of changes in HK-2 cells following exposure to nephrotoxic compounds of *Homo sapiens* GSE27211^2^ and it contains 438 samples. The data sets are ordered by size, and their results are presented accordingly.

### 3.2 GO Enrichment and Module Quality

The quality of the computed modules is evaluated by measuring their *enrichment* with respect to GO annotation, following a methodology that was established and used in previous works, among else in (Song et al., 2012; Hu and Zhao, 2016).

Concretely, for each computed module we perform a number of non-conditional hypergeometric tests using function hyperGTest of GOstats (Falcon and Gentleman, 2006). To be more specific, we note that GOstats provides an option to choose among three GO ontologies (“Biological Process”, “Cellular Component”, “Molecular Function”), and also an option to choose a “test direction”, i.e., checking for overrepresented or underrepresented terms. Collectively, there are six different ways of calling the non-conditional hyperGTest. We perform all these six tests on each module^2^.

These tests return a set of terms and corresponding *p*-values for each module. As usual, smaller *p*-values indicate a higher statistical significance. Following previous works (Song et al., 2012; Hu and Zhao, 2016), we keep the five smaller *p*-values for each module, and their geometric mean is viewed as measure of module quality.

More precisely, let *p*_*i*,*j*_ be the *i*th-order *p*-value calculated for module *j*. We define the quality of module *j* to be the negative logarithm of the geometric mean of the five best *p*-values for module *j*:

$$Q_{j}=-\left(\overset{5}{\underset{j=1}{\sum }}\log_{10}p_{i,j}\right)/5$$ (6)

### 3.3 Pipeline Evaluation and Comparison

#### 3.3.1 Average Cluster Quality

Following previous convention and methodology (Song et al., 2012; Hu and Zhao, 2016), we evaluate the performance of each pipeline by calculating an average module quality over all modules computed by the pipeline. More precisely, suppose that pipeline *x* outputs a number *n*_*x*_ of different modules. Then, given definition six the average module quality is defined as

$$\bar{Q}=\left(\overset{n_{x}}{\underset{j=1}{\sum }}Q_{j}\right)/n_{x}$$ (7)

**FIGURE 1:**
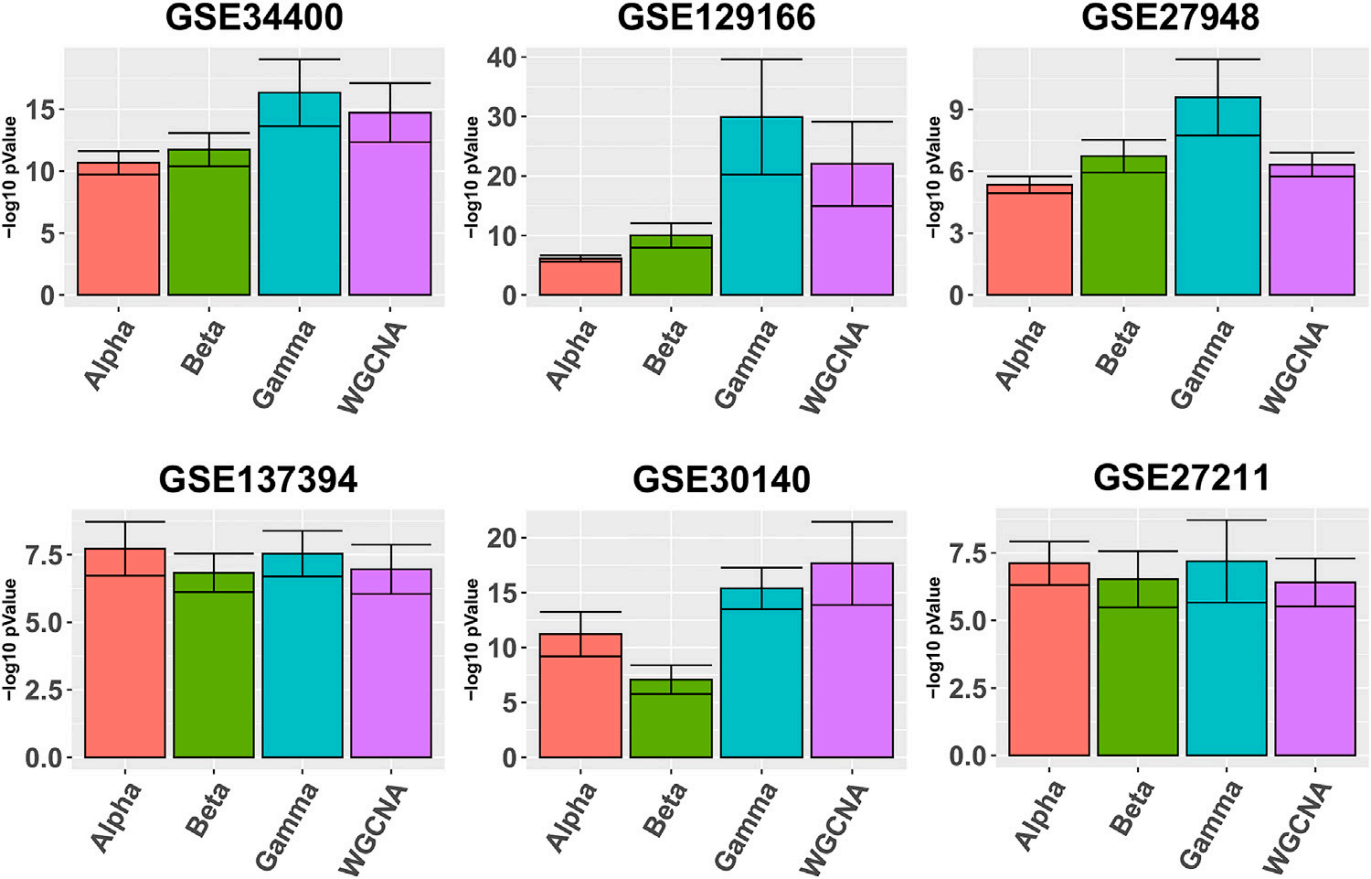
Gene ontology enrichment analysis comparing Alpha, Beta, Gamma with WGCNA in six real data sets. The five best GO enrichment *p*-values from all modules are log transformed, averaged and shown as barplots. Higher is better. Error bars indicate the 95% confidence intervals that have been calculated based on the standard deviation of the *p*-values.

Figure 1 depicts in bars the average quality $\bar{Q}$ (def. 7) for each pipeline. It can be seen that Gamma yields better average module quality in all six data sets, with the exception of GSE30140. In the same Figure we also observe that in half of the data sets Alpha outperforms Beta, and Alpha performs better on data sets with larger sample size.

#### 3.3.2 Ordered Cluster Quality

**FIGURE 2:**
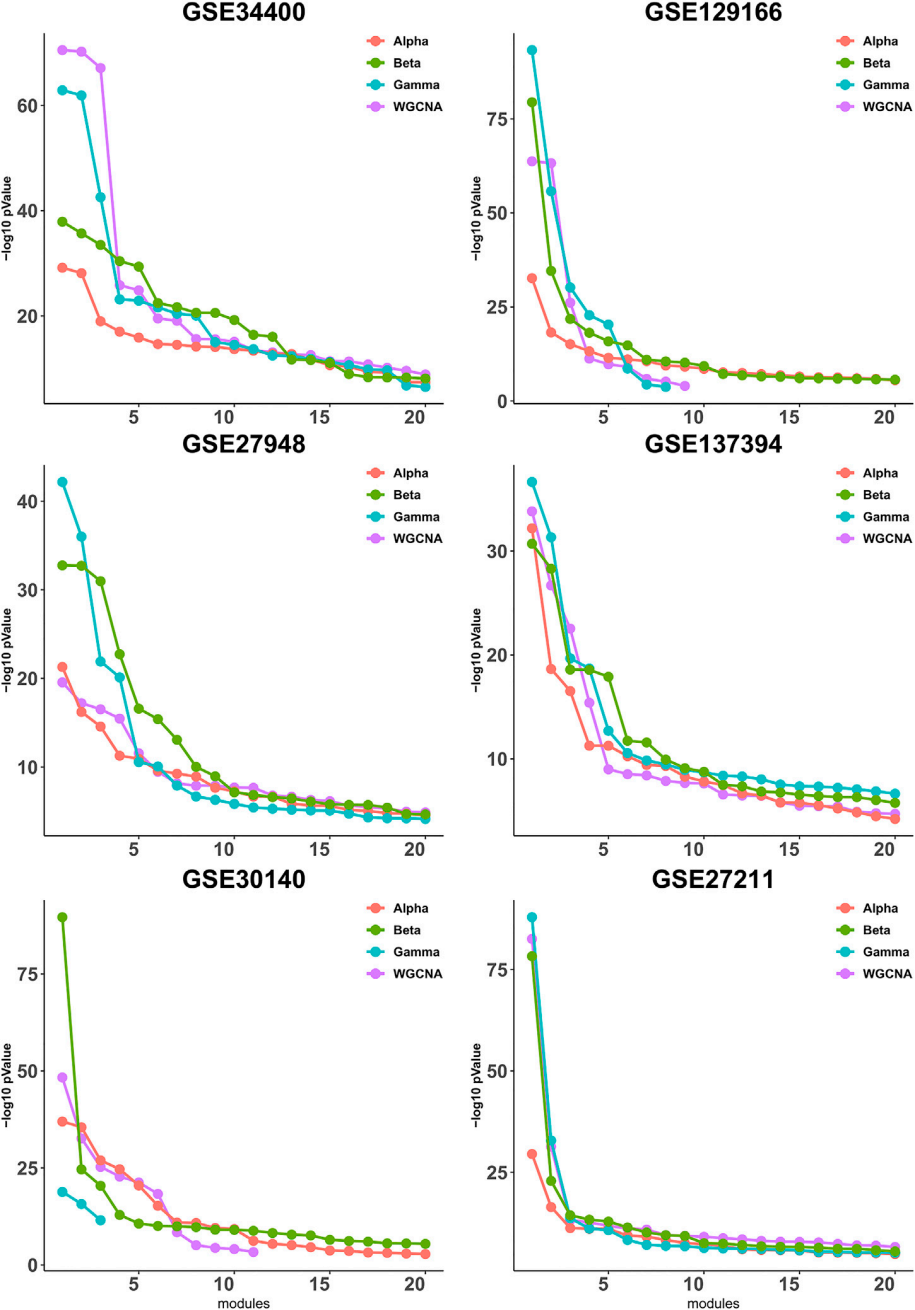
Gene ontology enrichment analysis of clusters produced by Alpha, Beta, Gamma with WGCNA in six empirical data sets. For each data set the sorted quality values (def. 6) of the modules are plotted. The *x*-axis and *y*-axis indicate the module indices and the module quality respectively.

It has been observed in (Gibbons and Roth, 2002) that expression-based clustering methods produce multiple clusters of relatively low enrichment. In view of this, we take a mode detailed look at the *p*-values for each module individually. To this end, we calculate the quality (as defined in Section 3.2) for each module, then sort the modules according to their quality and plot up to 20 corresponding values, whenever available. As shown in Figure 2, the difference between the four pipelines is more pronounced for the higher-quality modules and it becomes less clear for the lower-quality modules that are presumably less important from a biological point of view due to their lower quality.

**FIGURE 3:**
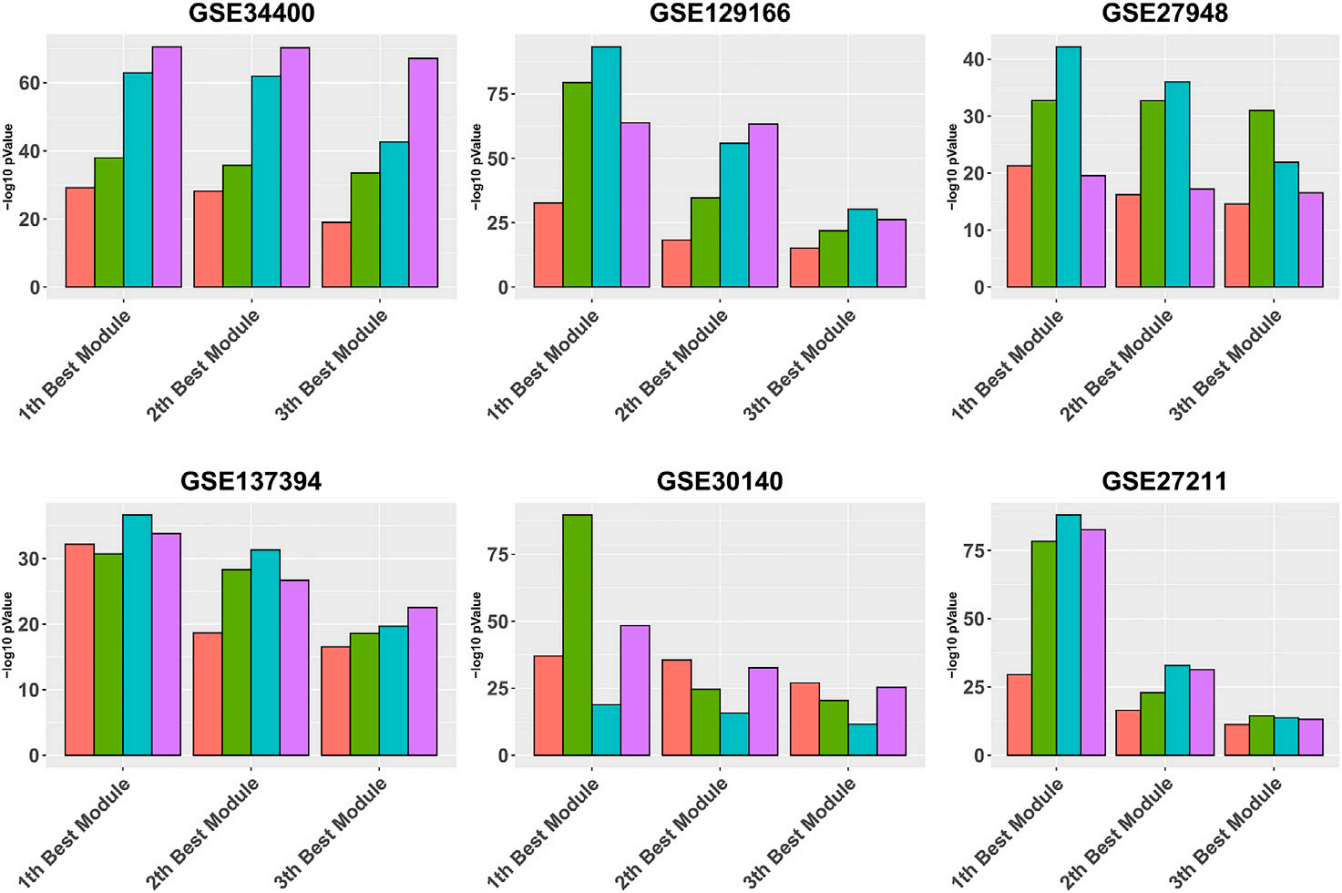
Gene ontology enrichment analysis of 10 best modules produced by Alpha, Beta, Gamma, and WGCNA in six real data sets. The mean over the five best GO enrichment *p*-values from the 10 top modules of each pipeline are compared. The *x*-axis and *y*-axis indicate the 10 best modules and the module performance respectively.

Figure 3 is similar to Figure 2, except that it focuses on the three best modules for each pipeline, for reading clarity. It can be seen that, in all data sets, Gamma returns the module with the highest enrichment, with the exception of GSE34400 and GSE30140. We note that GSE34400 has the least number of samples which is 44. In GSE30140, as discussed earlier, WGCNA is better on average (Figure 1) but even in this, case Beta produces a module of higher quality relative to WGCNA. Notably, in GSE30140, Beta’s top cluster is by far better than those of Gamma and WGCNA. This demonstrates a case where TOM leads to lower quality in the top module. The dominance of calibration-based methods, in general, extends to the order-2 module and, while still present in some data sets, diminishes in the order-3 module.

#### 3.3.3 Other comparisons.

Besides comparing the enrichment of the computed clusters, multiple other related questions can be considered. Here we perform two additional types of comparisons in order to demonstrate that the modules computed by the calibration-based methods can be significantly different than those computed by WGCNA.

The clustering algorithm used in WGCNA has a number of parameters that can affect the clustering outcome, but in this work, we use the default settings for all four pipelines. With these default settings, the algorithm rejects a number of trivial clusters of small size, and the corresponding genes do not appear in the clustering output. In Table 1 we wish to highlight the percentage of such genes that are not assigned to any module. In general, WGCNA leaves unassigned more nodes relative to calibration methods, and in particular Gamma. For example, in GSE30140, WGCNA ignores over 90*%* of the genes for the clustering, i.e. these genes are not included in any module; in comparison, Gamma assigns 82% of the genes to modules. We also observe that there is a significant variance in the number of clusters computed by the four pipelines and that WGCNA has a tendency to produce more clusters than Gamma (with the “slight” exception of GSE137394). These two facts combined imply that the sizes of the clusters computed by our pipeline are on average bigger than the standard WGCNA pipeline. The precise cluster sizes can be found along with the code and data in the public code repository. We also note that the very recent work in (Hou et al., 2021) has also identified the issue with unassigned genes in WGCNA, and introduced an additional clustering step that assigns all genes to an appropriately selected module, claiming higher module enrichment. The tendency of our pipeline to automatically do much of what (Hou et al., 2021) does in a “forced” way, is an interesting feature of our pipeline.

**TABLE 1:** A Clustering summary. The number of modules and percentages of unassigned genes for the four pipelines Alpha (A), Beta (B), Gamma (Γ), WGCNA (W).

Data set	Pipeline	# Clusters	% Of unassigned genes
GSE34400	A B Γ W	34 42 27 39	0.58 32.0 13.0 22.0
GSE129166	A B Γ W	60 35 8 9	0.01 15.5 2.13 6.50
GSE27948	A B Γ W	58 60 24 41	2.4 4.5 0.0 29.2
GSE137394	A B Γ W	29 53 48 40	0.8 62.3 56.00 74.1
GSE30140	A B Γ W	22 60 3 11	1.3 0.45 18.0 91.1
GSE27211	A B Γ W	31 66 51 84	0.56 50.0 32.0 38.13

Recall that in the computation of the quality measures, we kept the five GO terms with the smallest *p*-values for each module. In Table 2 we focus on the top module, and report how many of these five GO terms are shared between each pair of methods. We see that in two datasets (GSE129166 and GSE27211) the overlap between Gamma and WGCNA is significant (5 and 4 respectively). In other datasets, it can be as low as zero. This indicates that the computed clusters are potentially different (relative to WGCNA) in terms of their biological meaning and significance.

**TABLE 2:** Overlapping in the five GO terms of the top module for each pair of pipelines. Each table contains two data sets: the first data set is shown in the upper-triangular part of the table, and the second in the lower-triangular part. For instance, the number of GO terms shared between WGCNA and Gamma in GSE27211 can be found in the corresponding cell of the lower part of the third table (=4 in this case).

Data set		Alpha	Beta	Gamma	WGCNA
GSE34400	Alpha		3	1	0
	Beta	2		0	0
GSE129166	Gamma	4	3		0
	WGCNA	4	3	5	
GSE27948	Alpha		2	0	0
	Beta	0		0	0
GSE137394	Gamma	0	2		0
	WGCNA	0	0	2	
GSE30140	Alpha		5	0	0
	Beta	5		0	0
GSE27211	Gamma	0	5		4
	WGCNA	0	4	4	

## 4 Discussion

WGCNA is a widely used software package for identifying biologically meaningful clusters of genes. As highlighted in the title of the original work (Zhang and Horvath, 2005), WGCNA is in fact a versatile *general framework* that can be instantiated in multiple ways into concrete data-processing pipelines. The research community has adopted the GO enrichment of the computed modules as a proxy of the biological utility of a pipeline (Song et al., 2012; Hu and Zhao, 2016). Indeed, several research articles have been devoted to studying individual algorithmic components of WGCNA and their impact on GO enrichment, up until recently (Hou et al., 2021). For example, the current practice of using Pearson correlation as similarity measure for pairs of genes has been influenced by the outcome of an extensive study that considered various other similarity measures (Song et al., 2012).

In this work, we go beyond modifying the existing WGCNA components and propose an “architectural” change with the inclusion of a novel calibration layer that precedes the computation of pairwise similarities between the genes. The proposed calibration is a sigmoid transformation of the raw gene expressions that is applied separately to each gene. In addition, we replace Pearson correlation as similarity measure with an even simpler geometric measure (cosine similarity) that–somewhat curiously–has not been considered before, possibly due to “cultural” reasons related to the background of the research groups that undertook earlier efforts (Song et al., 2012). As discussed in Section 3, calibration appears to help the clustering algorithm capture modules with a higher average enrichment in Gene Ontology terms, with the effect being more pronounced for the modules of highest enrichment. It also appears to result in modules that can be qualitatively quite different than those computed by WGCNA.

Ultimately the biological utility of a specific pipeline can only be confirmed by applied biological discovery. While we are encouraged by our results in terms of the GO enrichment, we do not regard our methods as antagonistic to WGCNA but rather as alternatives that can be easily incorporated into existing WGCNA-based pipelines and hopefully provide an additional tool to biologists. For that reason, we provide code that can work directly with the existing WGCNA codebase.

### 4.1 Future Considerations

We wish to highlight an additional interesting fact. Topological Overlap (TOM), i.e., the formation of the final network based not on just pairwise similarities but also on second-order neighborhoods of the genes, appears to yield more enriched modules in our calibrated setting, as it has also been observed for other pipelines that are markedly different. This independent confirmation leads to the natural question of whether higher-order neighborhoods can enhance cluster quality as it has been observed recently in other types of datasets [e.g., see Qiu et al. (2017)]; we feel that this is a topic worth of more exploration. We have also found (although not reported in this paper) that dropping the scale-freeness step from our pipeline reduces module quality, as it does in the standard WGCNA pipeline. Interestingly, the single dataset (GSE30140) where TOM leads to a deterioration in module enrichment for the top module is also the only dataset where powering the network does not yield in practice a good fit to the scale-freeness criterion used by WGCNA. The notion of scale-freeness in biological networks has received significant criticism [e.g., see Broido and Clauset (2019)] and indeed the existence of datasets where scale-freeness is not present may provide a very interesting lead for further research on graph-theoretic alternatives to scale-freeness especially in terms of its synergy with topological overlap. We leave these questions open for future research.

## Data Availability

The datasets presented in this study can be found in online repositories. The names of the repository/repositories and accession number(s) can be found below: The public GitHub repository https://github.com/ikoutis/bioNets.

## Footnotes

1
https://www.ncbi.nlm.nih.gov/geo/query/acc.cgi?acc=GSE27948.

2
https://www.ncbi.nlm.nih.gov/geo/query/acc.cgi?acc=GSE27211.
